# Supplementary figures and images for: Correlation of drug resistance with single nucleotide variations through genome analysis and experimental validation in a multi-drug resistant clinical isolate of M. tuberculosis
Source: BMC Microbiol. 2020 Jul 25;20:223. doi: 10.1186/s12866-020-01912-6 (PMC7382824; doi:10.1186/s12866-020-01912-6)

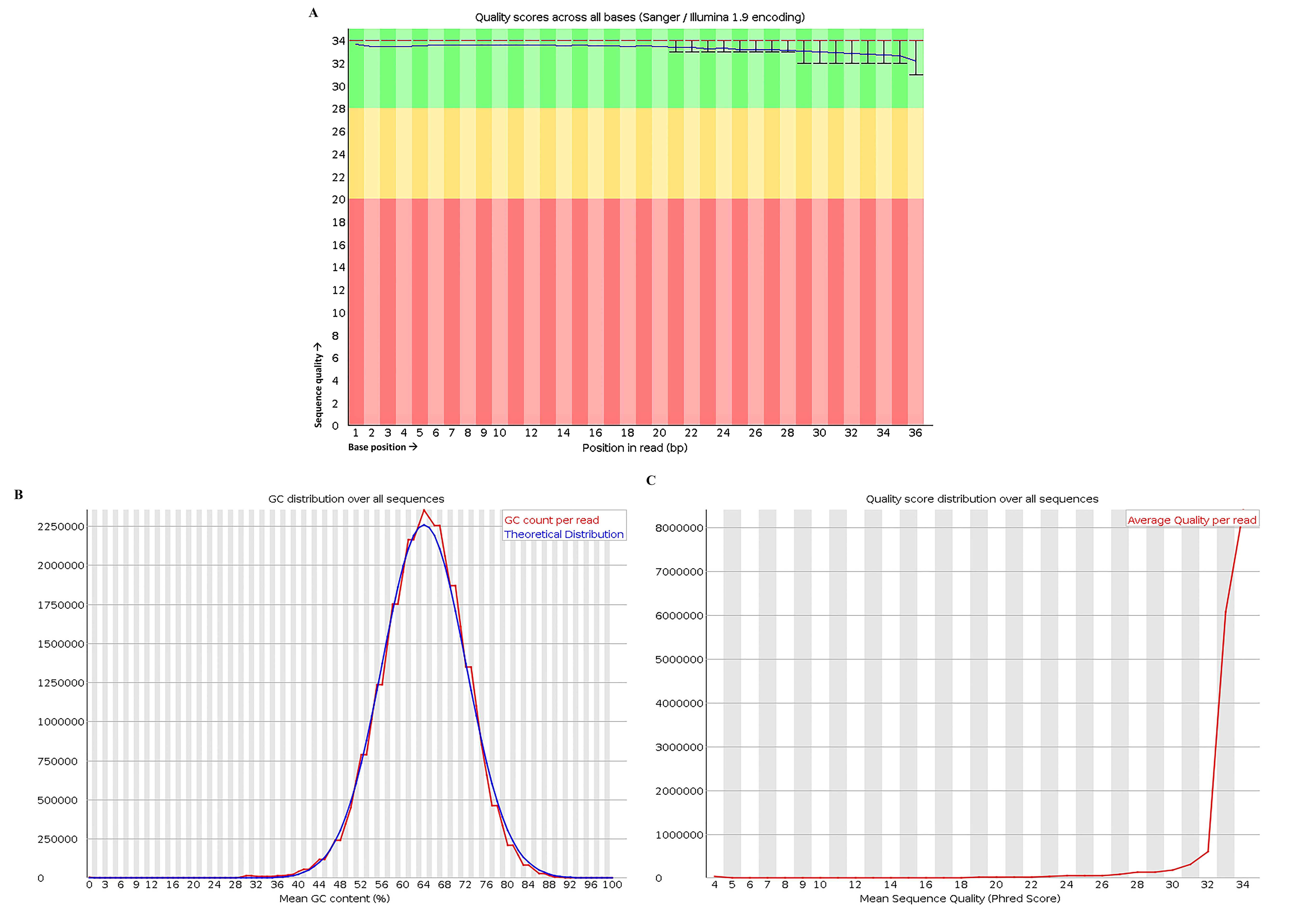

Supplement: Supplementary file 1 — Additional file 1. FastQC analysis. A. Assessment of quality of the sequence reads. The data shows quartile deviation with mean deviation (blue curve). Sequence quality is shown on the X axis and base position on Y axis. Green- high quality reads, yellow-average and red-low quality reads. B. Mean distribution of GC content, estimated from VPCI591 sequence data (red) compared to the theoretical distribution of the GC rich genome (blue). C. Mean sequence quality score, ranging from 32 to 34 indicating high quality reads. [file 12866_2020_1912_MOESM1_ESM.tif]

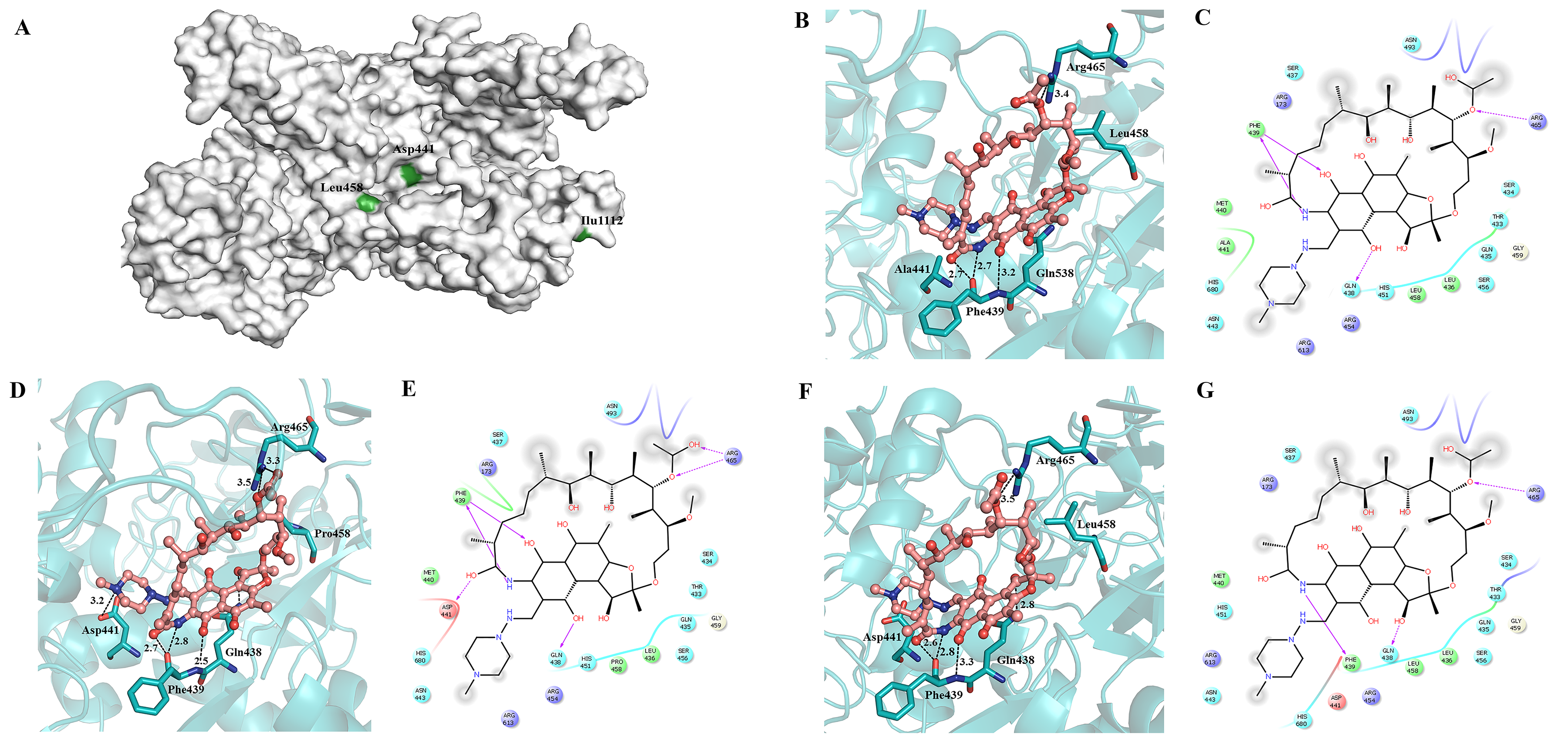

Supplement: Supplementary file 8 — Additional file 8. Docking analysis of SNV in RpoB. A. The crystal structure of RpoB (5UHB) is represented harbouring all the 3 variations, as present in VPCI591 in green. B, D and F represent the 3D view for D441A, L452P & I1112M respectively. Amino acid residues are shown with sticks and rifampicin (red) is shown with ball and stick model. Hydrogen bonds are shown as broken line (black). C, E and G represent the corresponding 2D sketch. Rifampicin is represented in black interacting to amino acids (colour circles) through purple arrows. [file 12866_2020_1912_MOESM8_ESM.tif]
